# Friction and durability of virgin and damaged skin with and without skin cream treatment using atomic force microscopy

**DOI:** 10.3762/bjnano.3.83

**Published:** 2012-11-08

**Authors:** Bharat Bhushan, Si Chen, Shirong Ge

**Affiliations:** 1 Nanoprobe Laboratory for Bio- & Nanotechnology and Biomimetics, The Ohio State University, 201 W 19th Ave., Columbus, OH 43210 USA; 2 Institute of Tribology and Reliability Engineering, China University of Mining and Technology, Xuzhou, Jiangsu 221116 China

**Keywords:** atomic force microscopy, damaged skin, pig skin, rat skin, skin cream

## Abstract

Skin can be damaged by the environment easily. Skin cream is an effective and rapid way to moisten the skin by changing the skin surface properties. Rat skin and pig skin are common animal models for studies and were used as skin samples in this study. The nano- and macroscale friction and durability of damaged skin were measured and compared with those of virgin (intact/undamaged) skin. The effect of skin cream on friction and durability of damaged and virgin skin samples is discussed. The effects of velocity, normal load, relative humidity and number of cycles were studied. The nanoscale studies were performed by using atomic force microscope (AFM), and macroscale studies were performed by using a pin-on-disk (POD) reciprocating tribometer. It was found that damaged skin has different mechanical properties, surface roughness, contact angle, friction and durability compared to that of virgin skin. But similar changes occur after skin cream treatment. Rat and pig skin show similar trends in friction and durability.

## Introduction

**Figure 1:**
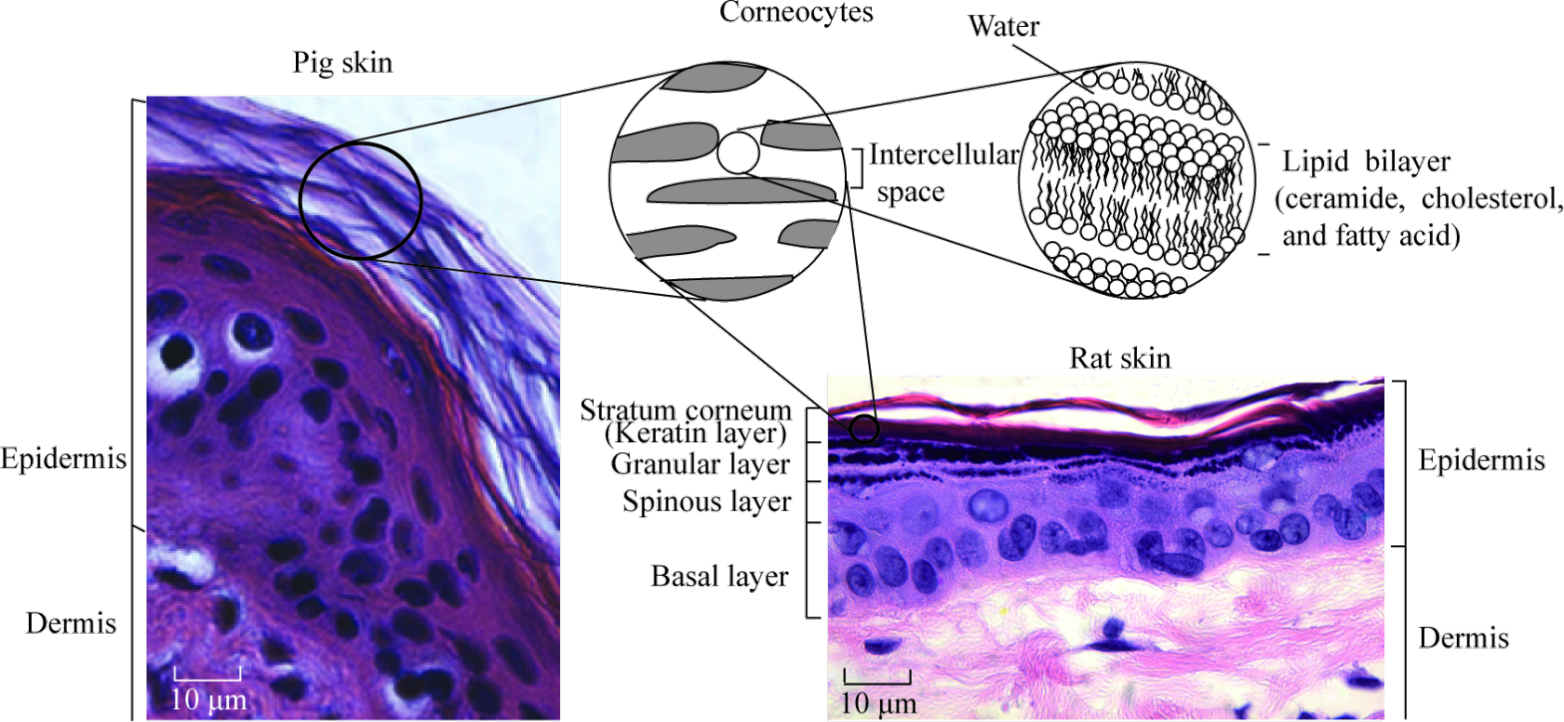
Histology of pig skin (photography reprinted from [7]) and rat skin (photography reprinted from [6]), and chemical structure of major lipid components of the stratum corneum.

Skin is the largest outer organ. The skin structure of mammals is mainly composed of three distinct layers: subcutis, dermis, and epidermis [1–6]. Rat skin and pig skin are common models used for skin in health and cosmetics studies. Figure 1 shows the epidermis and dermis of pig and rat skin [6–7]. The epidermis is the outer layer of skin. It contains four distinct cellular layers: basal layer, spinous layer, granular layer, and stratum corneum (keratin layer) [6]. Figure 1 also shows the chemical structure of the major components of the stratum corneum. Corneocytes with lipid bilayers exist in stratum corneum [6]. Intact (i.e., undamaged; hereafter referred to as “virgin”) skin is covered with a thin hydrophobic lipid layer in its outer layer, containing triglycerides, diglycerides, fatty acids, wax esters, squalene, cholesterol, and cholesterol esters [8]. The intercellular lipids play an essential role in the establishment or maintenance of water-retention capacity in the stratum corneum [9]. Skin supports the body with protection, sensation, heat regulation, water resistance and so on, but environmental conditions such as dry and cold weather can reduce the moisture content of skin and induce epidermal hyperplasia, mast cell degranulation, cytokine secretion, increased skin roughness and physical discomfort [6,10–11].

Skin care products are developed to moisturize skin surface by humectants, which are a component of skin creams that can increase the moisture retention of stratum corneum and reduce the incidence of dry and flaky skin in vivo [12]. Humectants in skin cream attract and hold water in the skin, acting on the inside (i.e., moisture from the dermis to the epidermis/stratum corneum) and on the outside (i.e., moisture from the environment to the skin) [13]. Glycerin, lactic acid, potassium lactate, urea, sodium PCA, and propylene glycol are the humectants in common skin creams [11]. In general, polyols are the most effective humectants, especially the trihydroxylated molecule glycerol. Moisturizers containing glycerol provide enduring moisturization by binding and retaining water or by minimization of water loss. Glycerol can also hinder crystal-phase transitions induced by humidity in stratum corneum lipids and thus enhance the function of the skin as a barrier. In healthy skin, as corneocytes migrate to the skin surface they mature from a fragile to a resilient phenotype. Envelopes of fragile corneocytes can be seen on exposed body parts such as the face, especially in winter [13]. When moisturizers are used, however, the corneocytes may still mature to the resilient phenotype. It has been demonstrated through in vivo studies that moisturizers containing glycerin promote the maturation of these corneocytes, probably by activating the residual transglutaminase activity retained within the stratum corneum [13]. Skin cream can smooth the skin surface and increase the hydrophilic properties by helping the stratum corneum restore the lost moisture and the regular packing of the lipid lamellae, as well as repair the function and improve the feel of the skin [14].

For the studies of aesthetic repair and percutaneous absorption of cosmetics and drugs, pig skin has been used [15–17]. In studies of the mechanical [18–21] and tribological properties [6,22] of skin, and the percutaneous absorption of cosmetics and drugs [23–24], rat skin has also been used as an animal model. Pig skin and rat skin have been compared for percutaneous absorption [25–26], epidermal barrier layer lipids and morphology [27–28]. Table 1 shows some surface characteristics of virgin rat and pig skin. The stratum corneum and epidermis of pig skin are thicker than those of rat skin. The pig skin has fewer hair follicles than rat skin. Pig skin has been reported to be the most suitable model for human skin because of its similar surface properties, such as body mass and skin-to-body surface-area ratio, sparse hair, thick epidermis, hair-follicle density, epidermal turnover kinetics, lipid composition and the biophysical properties of the lipids [27–28], and similar permeability, i.e., the fluxes through the skin and concentrations in the skin are of the same order of magnitude for both tissues [25–26].

**Table 1:** Comparison of selected surface characteristics between virgin rat and pig skin samples.

	virgin rat skin	virgin pig skin

RMS (nm)	148 ± 6	274 ± 10
nanohardness (MPa)	7 ± 1	19 ± 3
elastic modulus (MPa)	70 ± 7	91 ± 28
stratum corneum thickness (µm)	5.0 ± 0.8	12.3 ± 0.7
epidermis thickness (µm) [27]	21.7 ± 2.2	51.9 ± 1.5
number of hair follicles (per cm^2^) [27]	289 ± 21	11 ± 1

Understanding the differences between friction and durability of normal and damaged skin and the role of cream treatment of the damaged skin on friction and durability is of importance. Many studies have focused on the macroscale friction and durability of normal and damaged skin, but the skin properties are related to the nanoscale structures, and therefore an understanding of the nanoscale friction and durability is necessary. In this research, friction and durability of virgin and damaged skin were measured on the nanoscale to study the differences between them. The skin treated with skin cream was compared to untreated skin to study the effect of skin cream. The effect of velocity, normal load, relative humidity and number of cycles on friction was studied. Experiments were also carried out on the macroscale to study the scale effects. Both rat skin and pig skin were used as skin models in this study as they are common models for human skin. Surface roughness and mechanical properties are known to affect friction and these have been measured. A low contact angle is desirable for the adsorption of skin cream and has also been measured.

## Experimental

### Sample preparation

There were four categories of skin samples used in the tests: virgin skin, treated virgin skin, damaged skin, and treated damaged skin, for both rat and pig skin. The method to prepare virgin skin samples was described by Tang and Bhushan [6]. After the animal was sacrificed, the dorsal skin was immediately excised, subcutaneous tissues were scraped off with scissors, and the hair was shaved carefully with a razor. Then, the skin was gently cleaned with a 10% (v/v) soap solution (liquid hand soap, Kroger Co., Cincinnati, OH), rinsed with tap water for 30 s, and leveled on the table to dry under ambient conditions (22 °C, RH 25–35%). After that, the skin was rinsed with a commercial facial cleanser treatment, Clean & Clear Shine Control facial cleanser (Johnson & Johnson, Skillman, NJ). The facial cleanser was applied evenly on the skin surface with a cotton swab. Skin was lathered for 30 s and rinsed with tap water for 60 s. Then, the skin was leveled on the table and dabbed with filter papers (Whatman International Ltd., Maidstone, England) to remove excess water. After that the skin was cut into 10 mm × 10 mm samples and attached to the AFM sample pucks with rapidly drying glue. This virgin skin was considered to be the reference group.

A dry (damaged) skin can be realized by repeated skin wash with harsh soaps/detergents containing sodium lauryl sulfate (SLS) or by sodium dodecyl sulfate (SDS) surfactant [9,29–30], or by a 20 minutes treatment of the skin with acetone/ether (1:1), which causes removal of skin lipids and induces a chapped and scaly appearance. Scanning electron microscope studies of SDS-treated stratum corneum revealed selective depletion of the lipids from the intercellular spaces accompanied by marked disruption of multiple lamellae structures, and lipid analysis showed a considerable and selective loss of intercellular lipids such as cholesterol, cholesterol ester, free fatty acid, and sphingolipids [9,29]. Another approach is to use sticky cellophane tape to remove upper layers of skin, which also results in skin damage and scaly appearance after one day [31–32]. Tape stripping has been reported to produce results similar to treatment with a surfactant of a 5% aqueous solution of SDS under an occlusive dressing for 4 h [31]. In this study, SDS was chosen to prepare damaged skin without any inflammatory reaction accompanied by a significant decrease in its water-retention function. To produce a controllably damaged skin sample, a 5% weight aqueous solution of SDS, prepared by adding 5 g SDS (Bio-Rad Laboratories, Hercules, CA) into 100 g demineralized distilled water, was applied to the virgin skin surface by rubbing with a cotton swab for 30 seconds, and the skin was allowed to dry for 10 minutes, and the process was repeated.

Table 2 shows the composition of the skin cream (Vaseline Intensive Care Lotion, Unilever, Trumbull, CT) used in this study. Virgin skin and damaged skin were treated with common skin cream, which was rubbed over the entire skin surface for 30 s with a cotton swab. For the nanoscale tests 0.2 mg of commercial skin cream was applied to obtain a 150 nm film thickness. On the macroscale, 2 mg was applied forming a film of 1.8 µm thickness [6]. The same methodology was used both on rat and pig skin.

**Table 2:** Composition of common skin cream used in the study (based on manufacturer information).

skin cream	composition

common skin cream	water, glycerin, stearic acid, *helianthus annuus* seed oil, glycine soja, lecithin, tocopheryl acetate, retinyl palmitate, urea, collagen amino acids, sodium stearoyl lactylate, sodium isostearoyl lactate, mineral oil, sodium PCA, potassium lactate, lactic acid, petrolatum, dimethicone, avena sativa, keratin, glyceryl stearate, cetyl alcohol, methyl palmitate, magnesium aluminum silicate, fragrance, carbomer, stearamide AMP, triethanol amine, corn oil, methylparaben, DMDM hydantoin, disodium EDTA, BHT, propylene glycol, titanium dioxide

### Surface roughness and coefficient of friction measurements

Nanoscale surface roughness and coefficient of friction were measured by using a commercial AFM system (Dimension Nanoscope IIIa, Veeco, Santa Barbara, CA) under ambient conditions. Fort A-20 tips (Si, N-type, 10 nm radius, spring constant of 3 N/m) (Appnano, Santa Clara, CA) were used. The coefficient of friction was calibrated by the method described by Bhushan [33]. The friction force measurements were made over a scan length of 10 µm and at a scan rate of 1 Hz at various increments of normal load ranging from 25 nN to 250 nN. By plotting the friction force as a function of the normal load, an average value of the coefficient of friction was obtained from the slope of the fitted line of the data.

The macroscale coefficient of friction was measured by using a POD reciprocating tribometer, with measurement techniques described in detail by Bhushan [34–35]. The tests were carried out in an ambient environment over a stroke length of 10 mm and at a velocity of 0.4 mm/s and at a normal load ranging from 20 mN to 60 mN, unless otherwise noted. A sapphire ball with a 1.5 mm radius and surface roughness of about 2 nm RMS was fixed in a stationary holder. The normal load and friction force were measured with the semiconductor strain gages mounted on a crossed-I-beam structure. By plotting the friction as a function of normal load, an average coefficient of friction was obtained from the slope of the fitted line of the data. For each property, a minimum of six measurements was made. The ±1σ values were presented in the data plots.

### Effect of velocity and normal-load measurements

To study the effect of velocity on nanoscale friction, experiments were carried out using the AFM by changing the scan frequency from 0.1 to 50 Hz while the scan size was maintained at 10 µm, which allowed a range of velocity from 2 to 1000 µm/s. For the effect of normal load experiments, the normal load was varied from 50 to 750 nN at a 10 µm scan length and a scanning velocity of 20 µm/s. The macroscale experiments were performed by using a POD reciprocating tribometer. To study the effect of velocity, the velocity was varied from 0.4 to 4 mm/s over a stroke length of 2.5 mm at the normal load of 50 mN. For the effect of normal load experiments, the normal load was varied from 10 to 50 mN over a stroke length of 2.5 mm at a velocity of 0.4 mm/s.

### Effect of relative humidity measurements

A homemade humidity-control chamber system [36] was used to study the effect of relative humidity on the friction and durability of skin samples. A humidity detector was used to monitor the humidity inside AFM chamber. The relative humidity ranged from 4 to 95%. The skin sample was placed at each humidity value for about 1 h prior to the test.

### Durability measurements

The durability measurements were carried out by repeated cycling tests. The nanoscale durability tests were carried out by using the AFM at a velocity 20 µm/s and at a normal load of 250 nN for 3600 cycles. The macroscale durability tests were performed by POD reciprocating tribometer with a velocity 1 mm/s and at a normal load of 50 mN for 3000 cycles.

### Contact-angle measurements

The apparent contact angles were measured for various samples. Measurements were made with a Rame-Hart automated goniometer model 290-F4, where 5 μL water droplets were deposited onto the sample surface and the contact angle was measured.

### Nanoindentation measurements

The nanoindentation measurements were made by using a Hysitron Triboscope (Hysitron Inc., Minneapolis, MN) in the constant displacement rate loading mode with a three-sided pyramidal diamond (Berkovich) tip. In this study, the maximum indentation displacement was controlled to be 1000 nm [6]. The method for the hardness (*H)* and the elastic modulus (*E)* determination was based on established methods [37–38]. Briefly, *H* was calculated from

[1]
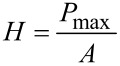


where *P*_max_ is the maximum imposed load, and *A* is the projected contact area. The relationship between the contact area and the contact depth was obtained from calibrating the tip with a standard material of known mechanical properties such that *A* is readily obtained from the load–displacement data.

*E* was analyzed according to the following equations:

[2]
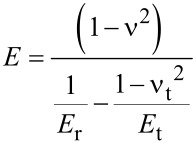


where *E*_t_ and ν_t_ are the elastic modulus and the Poisson’s ratio of the indenter tip respectively; ν is the Poisson’s ratio of skin assumed to be 0.5 [6]; *E*_r_ is the reduced modulus given as follows:

[3]
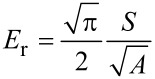


where *S* is the contact stiffness obtained from the slope of the unloading curve.

## Results and Discussion

The nanoindentation properties are presented in the first section. Then the surface roughness, contact angle and nano- and macroscale friction data of rat skin are presented. Finally, data of pig skin are presented.

### Nanoindentation properties of rat and pig skin

**Figure 2:**
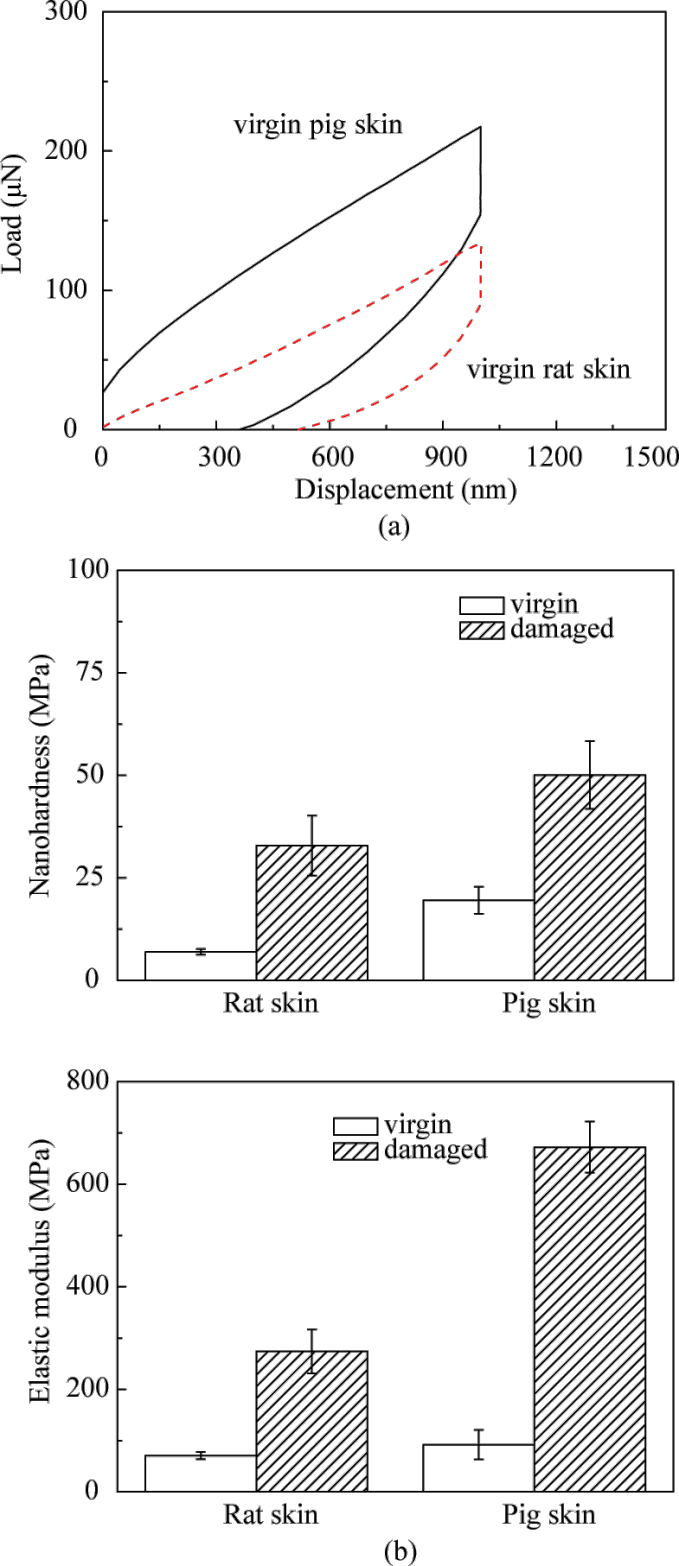
(a) Load–displacement curves and (b) nanohardness and elastic modulus data for rat and pig skin.

Mechanical properties of rat and pig skin were measured by using a nanoindenter. The load–displacement curves for rat and pig skin are presented in Figure 2a. Under the same displacement control, the load required for a given displacement for pig skin is larger than that for the rat skin, which means the pig skin is harder than rat skin. The nanohardness and elastic modulus data are presented in Figure 2b. Table 1 summarizes the mechanical properties data for virgin pig and rat skin. Both the nanohardness and elastic modulus of pig skin samples are higher than those of rat skin samples, and those of the damaged skin are higher than virgin skin for both rat and pig skin. The differences between the damaged skin and virgin skin for pig skin are greater than those for rat skin.

### Surface roughness, contact angle and friction properties of rat skin

#### Surface roughness, contact angle and nanoscale friction

**Figure 3:**
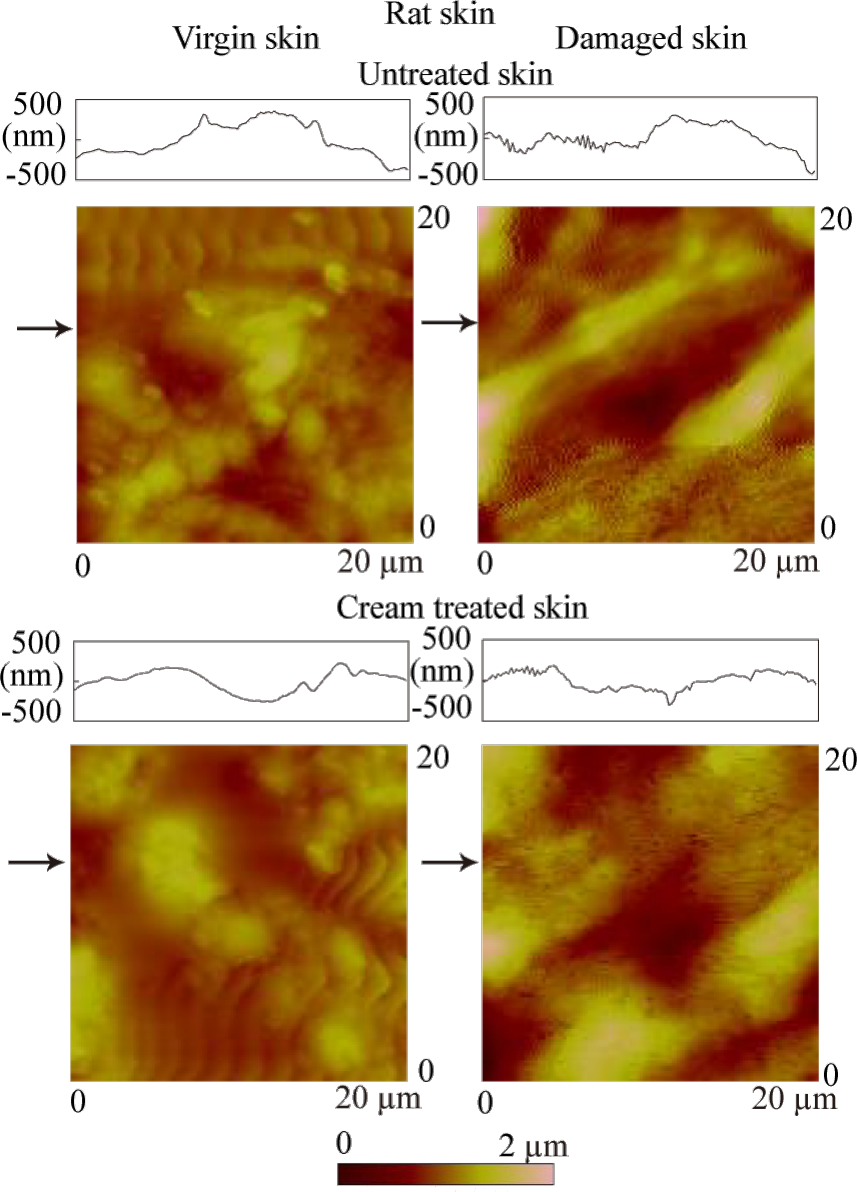
AFM topography maps and roughness profiles taken at arrows indicated for virgin rat skin, damaged skin, treated virgin skin and treated damaged skin.

**Figure 4:**
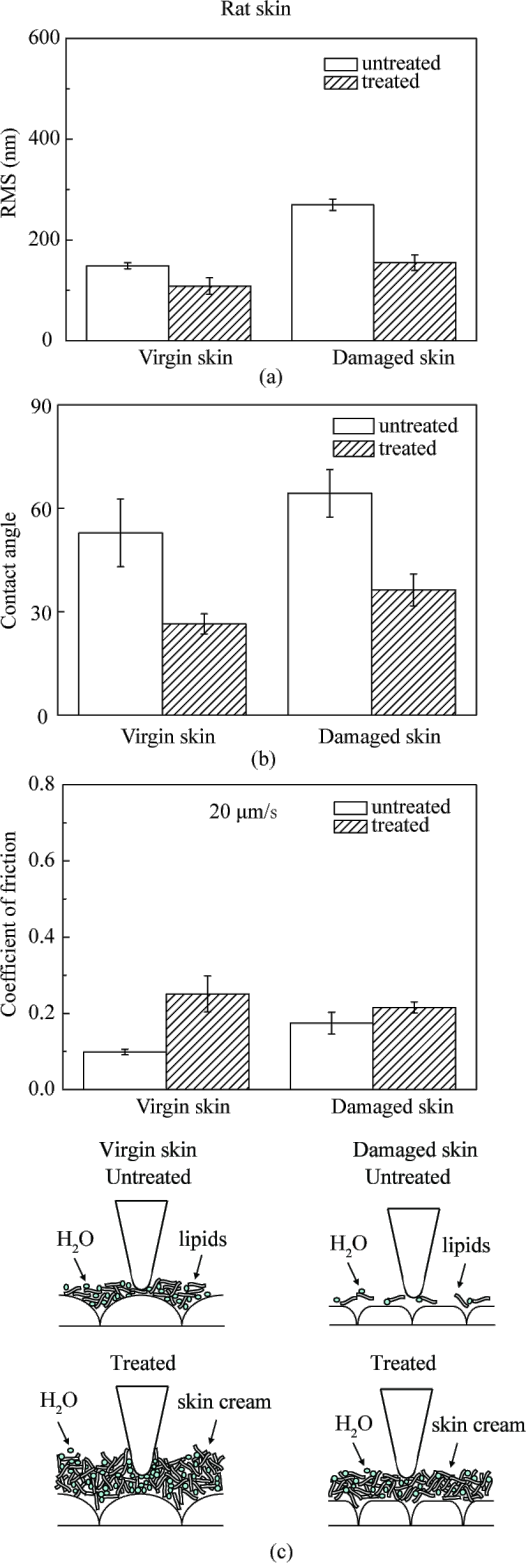
(a) RMS roughness, (b) contact angle and (c) coefficient of friction on nanoscale and schematic cartoons of the tip–skin-cream interaction of virgin rat skin, damaged skin, treated virgin skin and treated damaged skin.

Figure 3 shows topography maps and corresponding height profiles of the cross section indicated by the arrows on a 20 µm × 20 µm scan size for virgin skin, damaged skin, cream-treated virgin skin and cream-treated damaged skin. The height profiles appear smoother for virgin skin compared with damaged skin, and for cream-treated skin compared with untreated skin. The RMS roughness data, which serve as quantified expressions of the surface characteristics, are shown in Figure 4a. The damaged skin has a higher roughness than virgin skin. After treatment with skin cream, the roughness of virgin skin and damaged skin decreased. The reasonable explanation is that the skin cream can fill the gap between the cells of stratum corneum.

The contact angle data for virgin skin and damaged skin are shown in Figure 4b. The contact angle of virgin skin is lower than damaged skin due to physical and chemical changes to the skin surface. An increasing surface roughness may be partially responsible for an increase in contact angle of the damaged skin [39]. After treatment with skin cream, the contact angles of virgin and damaged skin decreased. The hydrophilic groups in skin cream, such as hydroxyl group, amines group, and carboxyl group in the humectants, increase the surface hydrophilicity and lead to a lower contact angle.

**Figure 5:**
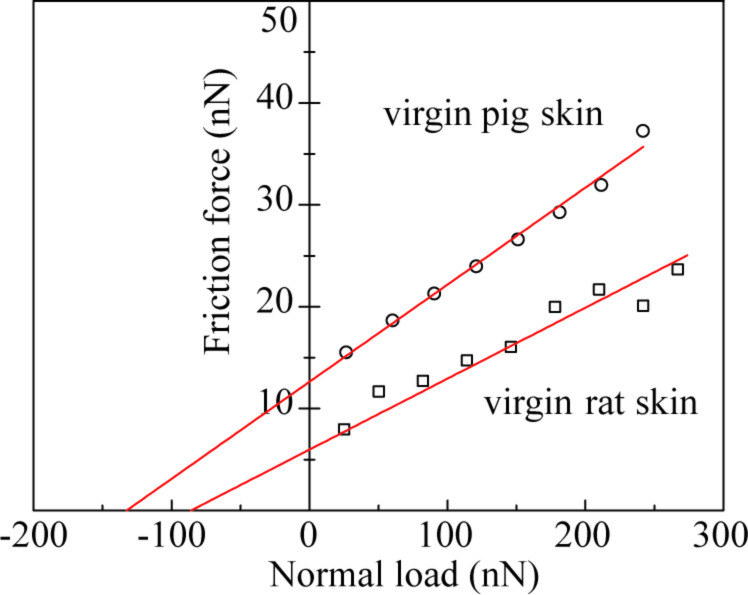
Friction force as a function of normal load curves for virgin rat and pig skin.

Figure 5 shows curves of friction force as a function of normal load for virgin rat and pig skin. An average value of coefficient of friction was obtained from the slope of the fitted line of the data. The intercept on the horizontal axis of normal load is the adhesive force, which is dominated by the meniscus contribution. Figure 4c presents the coefficients of friction of various skin samples. The coefficient of friction of damaged skin is higher than that of virgin skin, and increases for both virgin and damaged skin after treatment. Schematics show various rat skin interfaces. Damage to skin results in greater surface roughness and shrinking of the stratum corneum cells due to water loss, which hence increases the number of asperities on the surface [35]. The natural lipids present also deplete. Cream treatment for both skin types increases friction. Liquid films (lipid and condensed water vapor) present on the skin surface reduce the interfacial shear strength leading to lower friction; however, a thicker film forms meniscus bridges at asperity contacts leading to higher friction [34–35,40]. Cream treatment moistens and softens the skin, which leads to a greater ductility and larger real area of contact. Larger contact area and formation of meniscus bridges are responsible for higher friction in cream-treated skin [6].

#### Effect of velocity, normal load, relative humidity and number of cycles on nanoscale friction

**Figure 6:**
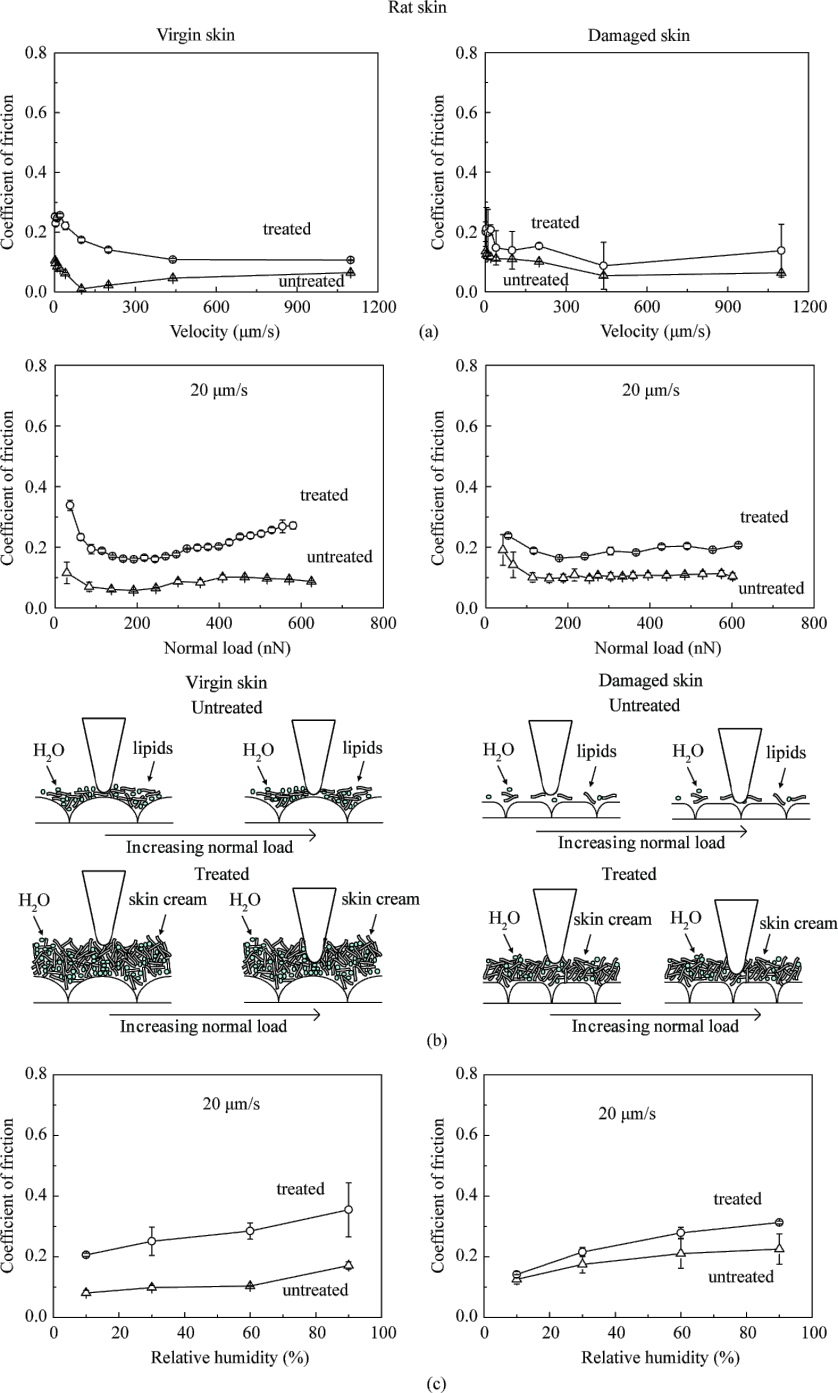
Effect of (a) velocity, (b) normal load, and schematic cartoons of tip–skin interaction, and (c) effect of relative humidity on the coefficient of friction on the nanoscale for virgin rat skin, damaged skin, treated virgin skin and treated damaged skin.

Figure 6a shows the coefficient of friction as a function of velocity for various skin samples. The data shows that friction decreases with an increase of velocity for all skin samples. At low velocity, the friction is dominated by meniscus force as proposed by Tang and Bhushan [6]. The tip sliding results in shearing and reformation of meniscus bridges. As the velocity increases, the meniscus bridges cannot be fully reformed, resulting in a drop in adhesive force and coefficient of friction. In the case of cream-treated skin, the skin cream is typically a shear-thinning fluid, and the viscosity decreases with the increasing shear rate leading to a decrease in the coefficient of friction [6,36].

Figure 6b shows the coefficient of friction as a function of normal load. The data shows that the friction for untreated skin samples first decreases then levels off, whereas, for the treated skin samples, it first decreased then increases above a certain load. As the tip moves towards the sample, a sudden mechanical instability occurs, and the tip jumps into contact with the film and a meniscus bridge is formed. But the tip does not slide in a steady manner on the surface at low normal load, and it may get rid of the meniscus bridges and bounce leading to a high deflection of the tip resulting in high friction data at the beginning. At higher load, the tip penetrates into film and slides in a steady manner, and the meniscus force dominates the friction. The coefficient of friction of treated skin samples increases above a certain load. It is believed that at larger load, the tip penetrates into the thick film and the formation of large meniscus bridges provides additional resistance responsible for the increasing friction [41–42].

Figure 6c shows the coefficient of friction as function of relative humidity. As the relative humidity increases, the coefficient of friction of all untreated and treated skin samples increases. As discussed earlier, the hydrophilic groups in the humectants of skin cream tend to form hydrogen bonds with water molecules, such that the humectants help the skin surface to attract water molecules in the environment, which increases the adhesive force leading to increasing coefficient of friction especially at high humidity [6]. Due to the hydrophobic lipid layer of virgin skin and some still present on the damaged skin, water hardly absorbs or penetrates into the skin surface, and humidity has less effect on it.

**Figure 7:**
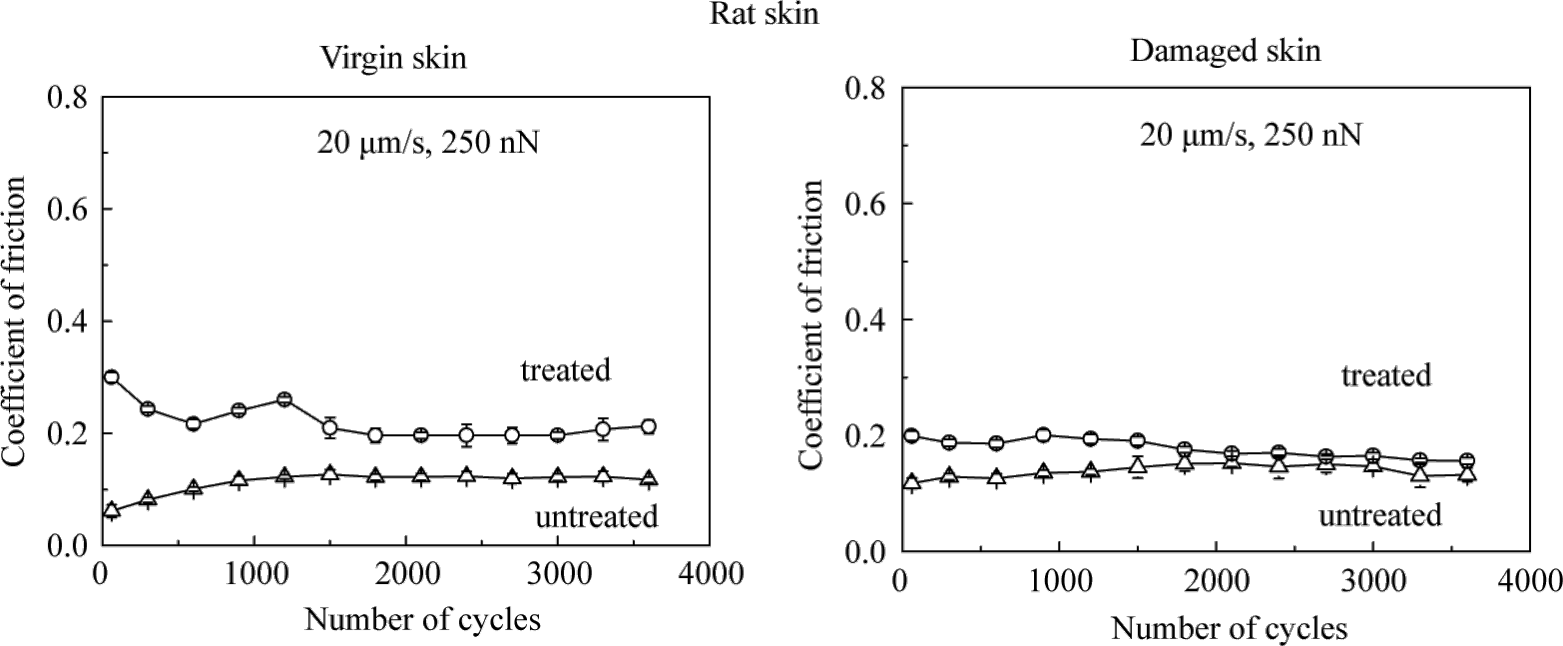
Effect of the number of cycles on the coefficient of friction on the nanoscale for virgin rat skin, damaged skin, treated virgin skin and treated damaged skin.

For durability studies, the friction experiments were performed by cycling the tip over the samples. Figure 7 shows the effect of the number of cycles on various skin samples. For untreated virgin and damaged skin, the coefficient of friction in the initial cycles is related to the removal of the thin lipid film on the skin surface, and then remains constant because the interaction between skin cream, skin surface, and environment reaches equilibrium. For cream-treated skin, the coefficient of friction decreases with the increasing number of cycles. This is believed to be caused by the change of cream film thickness. When cream is first applied to the skin surface, the cream cannot be absorbed immediately by the skin, and the cream liquid accumulates at the contact interface, resulting in a larger liquid height and greater viscous drag to motion. However, after several scans, because of the absorption of the skin cream and the evaporation of the water content, the cream film thickness decreases, which is responsible for the decrease in adhesive force and coefficient of friction. The skin cream finally covers the skin surface as a stable gel network (surfactant, fatty amphiphile, and water) and friction remains constant [6].

#### Macroscale friction and the effect of velocity, normal load and number of cycles

**Figure 8:**
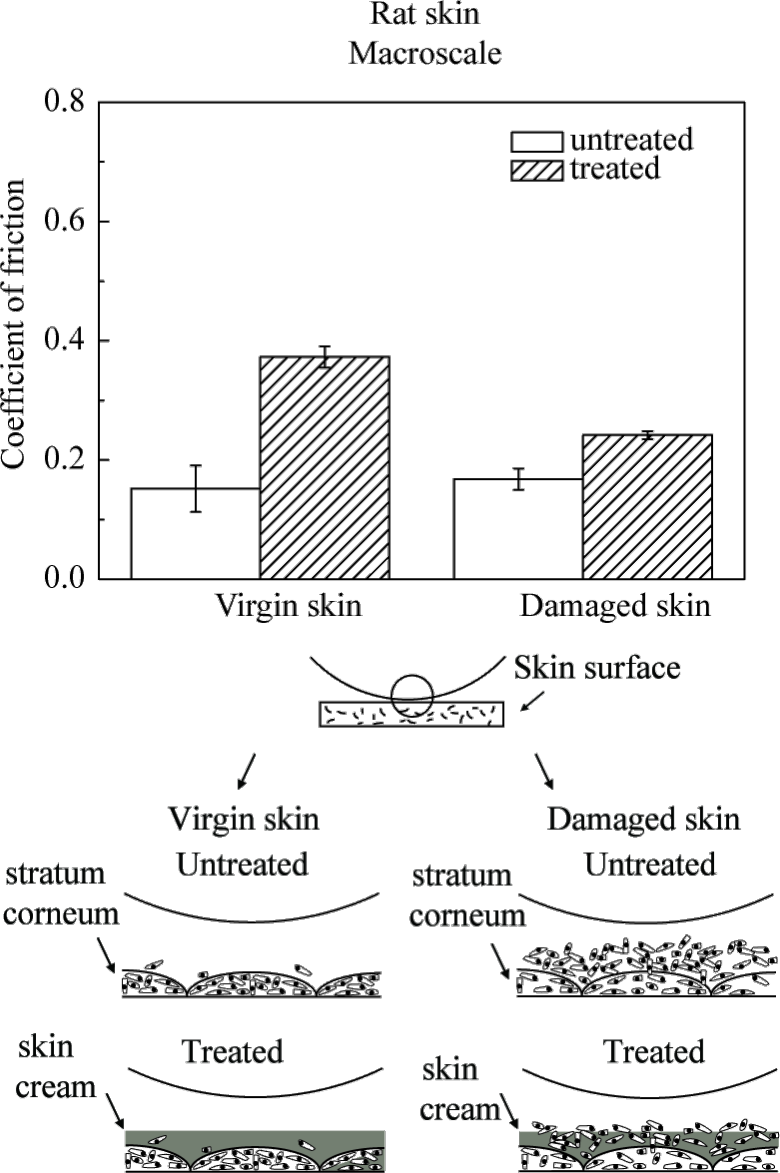
Coefficient of friction and schematic cartoons of the tip–skin interaction on macroscale for virgin rat skin, damaged skin, treated virgin skin and treated damaged skin.

Macroscale data for coefficient of friction for various skin samples is shown in Figure 8. The coefficient of friction of damaged skin is comparable to virgin skin. For damaged skin, as discussed earlier, the levels of the fragile corneocytes generally increase, so the stratum corneum of damaged skin is torn rapidly at high loads in macroscale experiments forming a lubricant layer between the tip and the skin surface, which is more easily sheared, and may compensate the loss of the lipid layer. After the application of skin cream, the skin surface properties change, and the skin is moistened and softened by the skin cream, which leads to a greater ductility and a larger real area of contact resulting in stronger adhesion, such that the coefficient of friction of cream-treated skin is higher than that of virgin skin [6].

**Figure 9:**
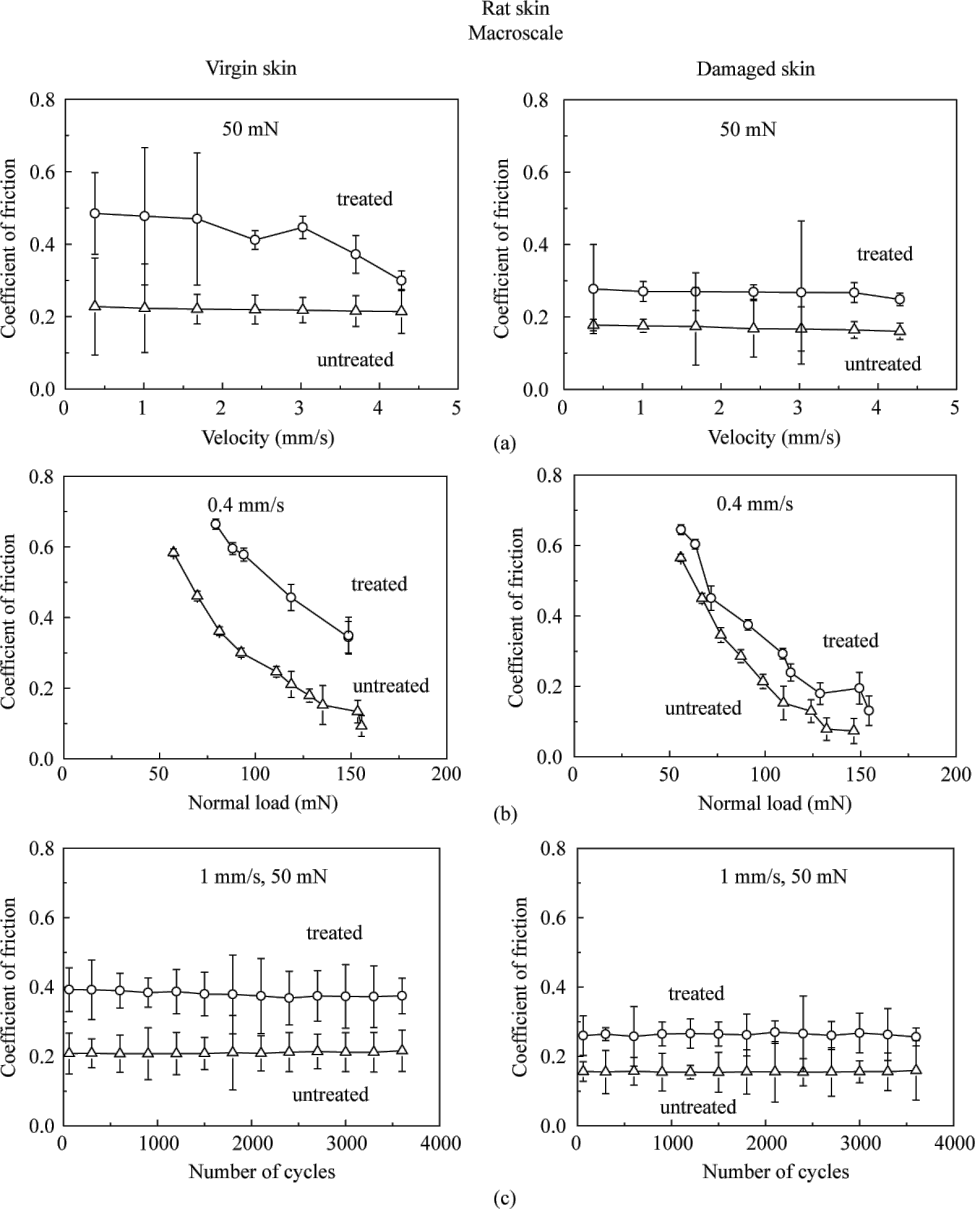
Effect of (a) velocity, (b) normal load, and (c) number of cycles on the coefficient of friction on macroscale for virgin rat skin, damaged skin, treated virgin skin and treated damaged skin.

Figure 9 presents the effect of velocity, normal load and number of cycles on the macroscale friction. Figure 9a shows that the coefficient of friction decreases as the velocity increases. The treated skin samples show a greater change than untreated skin samples. The reduction is similar to that on the nanoscale, since skin cream is a shear-thinning fluid as mentioned earlier.

Figure 9b shows that the coefficient of friction decreases as the normal load increases. Increased surface roughening and a large quantity of wear debris are believed to be responsible for the decrease of friction with an increase of normal load [40]. Asperity deformation of skin is primarily elastic, and as the normal load increases, elastic deformation at the asperities is large, such that the individual asperities on the contacting surface are totally deformed, and the contact region approximates to the contact of a large single asperity [35]. In this case, μ 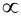 *W*^−1/3^, and the coefficient of friction μ decreases with the increase of normal load *W* [6]. Figure 9c shows that the coefficient of friction remains almost constant on the macroscale for the four skin samples with the number of cycles, which suggests little damage during the cycling test.

### Surface roughness, contact angle and friction properties of pig skin

#### Surface roughness, contact angle and nanoscale friction

**Figure 10:**
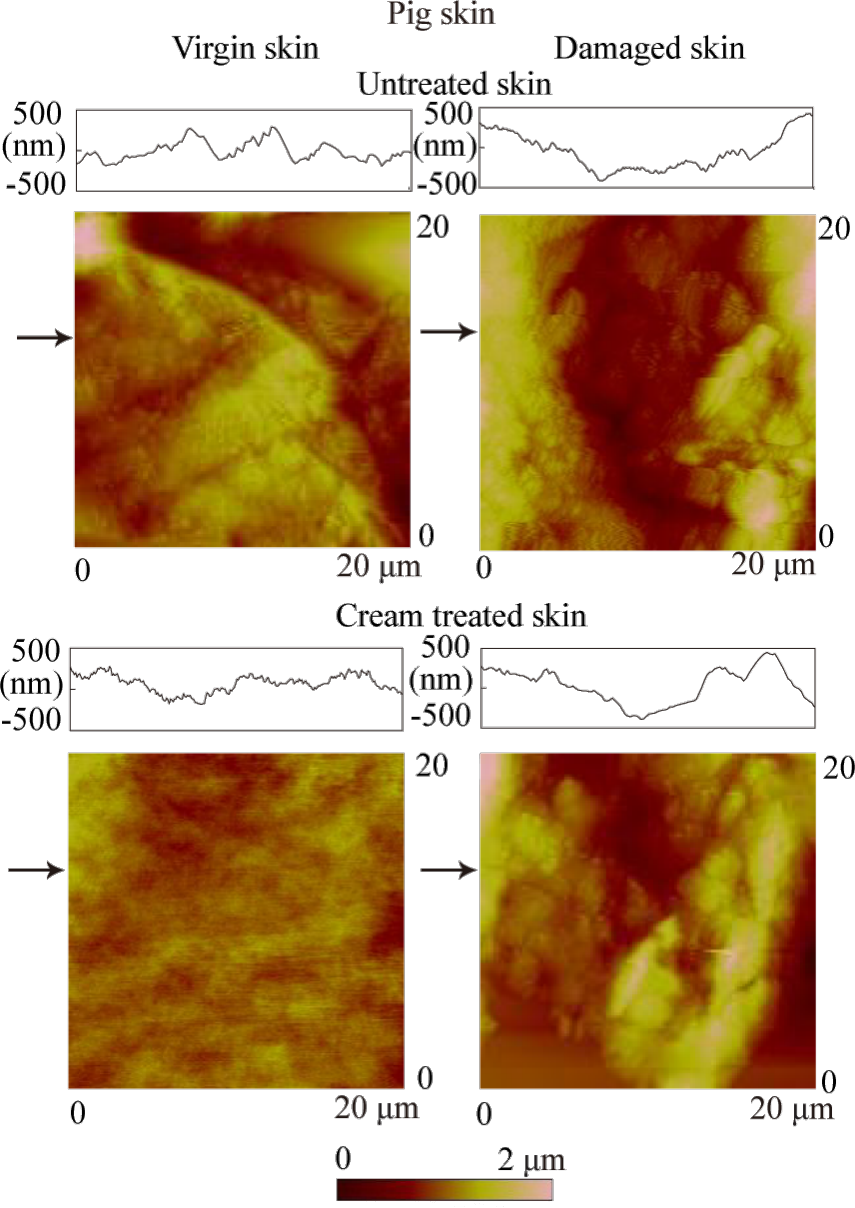
AFM topography maps and roughness profiles taken at arrows indicated for virgin pig skin, damaged skin, treated virgin skin and treated damaged skin.

**Figure 11:**
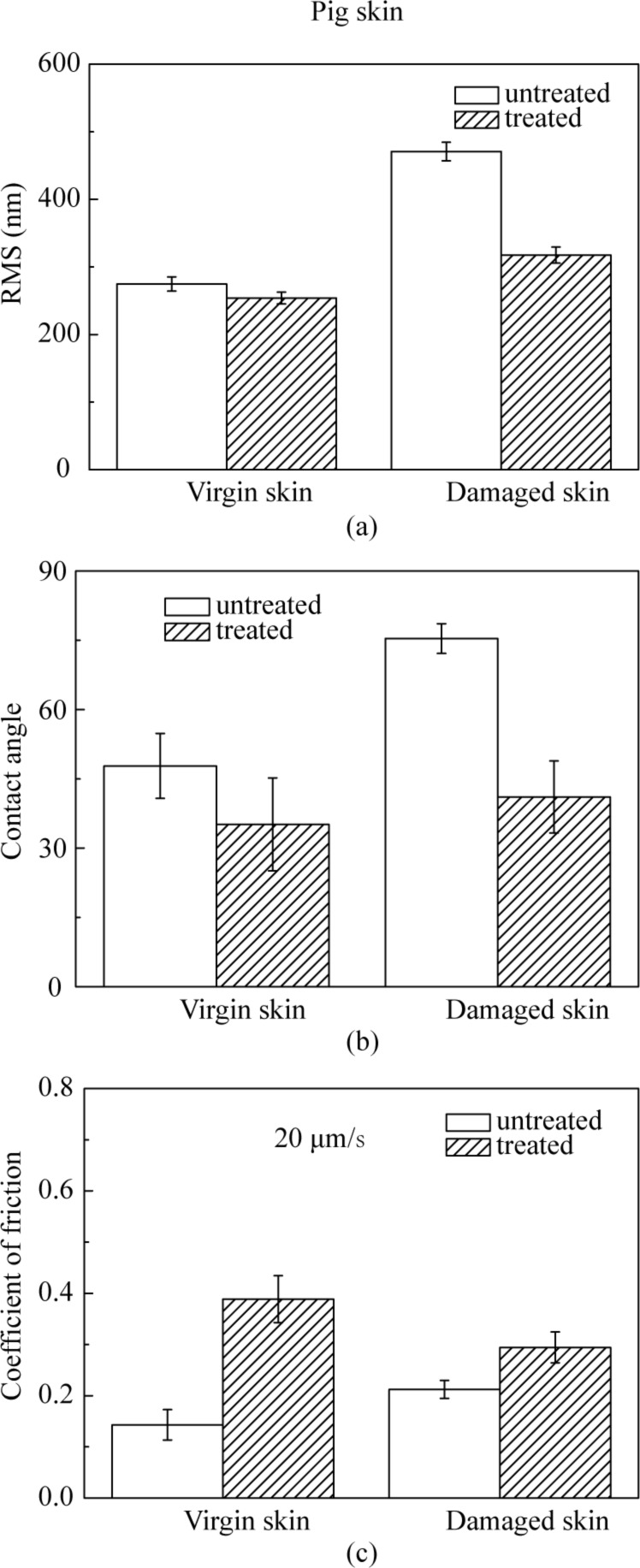
(a) RMS roughness, (b) contact angle, and (c) coefficient of friction on the nanoscale of virgin pig skin, damaged skin, treated virgin skin and treated damaged skin.

Figure 10 shows AFM topography on a 20 µm × 20 µm scan size for virgin pig skin, cream-treated virgin skin, damaged skin and cream-treated damaged skin. Figure 11a shows that the damaged skin has a higher surface roughness than virgin skin, i.e., the same trend as for rat skin, but the difference between virgin and damaged pig skin is more distinct than that for rat skin. After treatment, the roughness of both virgin and damaged skin decreased. As shown in Figure 11b, the contact angle of damaged skin is higher and decreases after treatment with skin cream, as observed earlier for rat skin.

Figure 11c shows the coefficient of friction of various skin samples. The coefficient of friction of damaged skin is higher than virgin skin. After treatment, the coefficient of friction of virgin and damaged skin increases. The coefficient of friction of pig skin is higher than that of rat skin because of the different surface characteristics discussed earlier.

#### Effect of velocity, normal load, relative humidity and number of cycles on nanoscale friction

**Figure 12:**
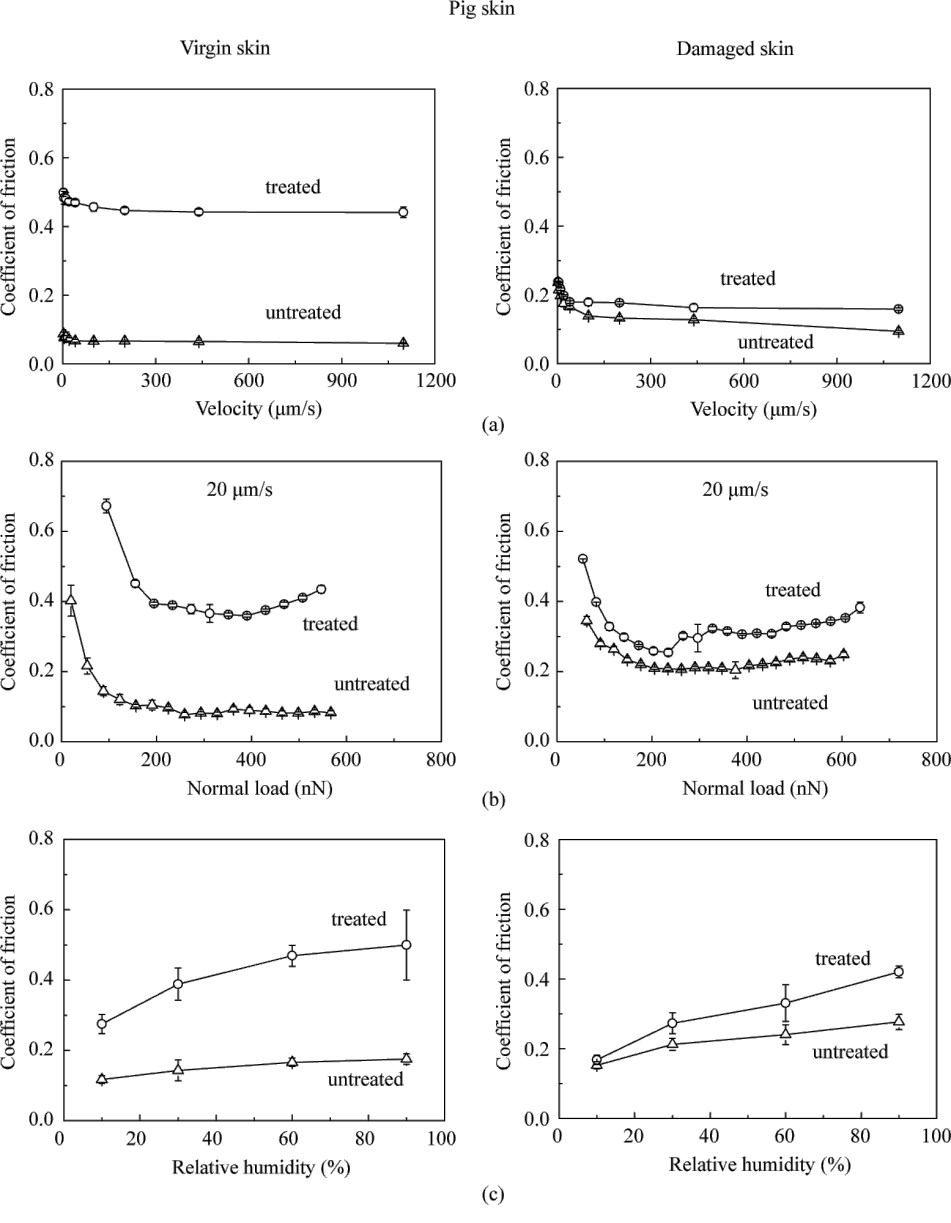
Effect of (a) velocity, (b) normal load and (c) relative humidity on the coefficient of friction on the nanoscale for virgin pig skin, damaged skin, treated virgin skin and treated damaged skin.

Figure 12 shows the effect of velocity, normal load and relative humidity on various skin samples. The coefficient of friction slightly decreases initially with an increase of velocity; the decrease is significant with an increase in normal load. It increases as the relative humidity increases. The trends are the same as those for rat skin.

**Figure 13:**
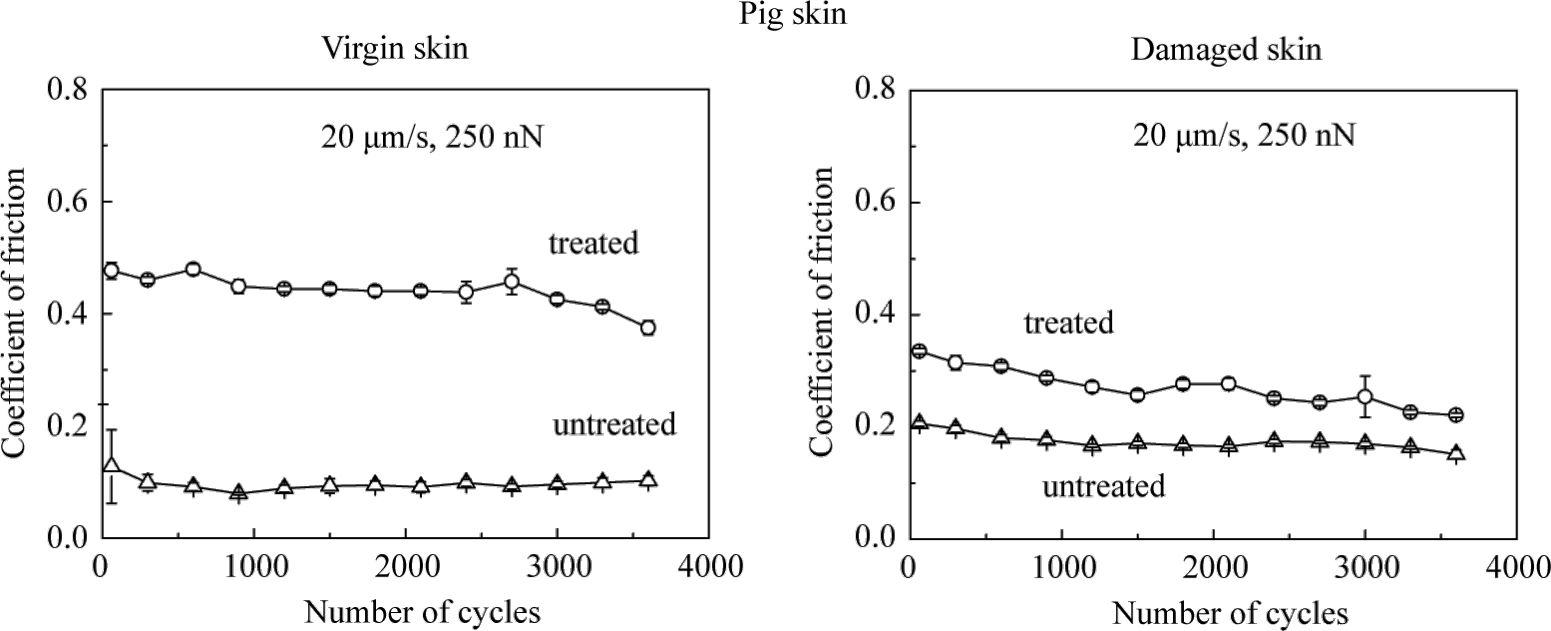
Effect of the number of cycles on the coefficient of friction on the nanoscale for virgin pig skin, damaged skin, treated virgin skin and treated damaged skin.

Figure 13 shows the effect of the number of cycles on various skin samples. The coefficient of friction of treated pig skin samples shows a greater decrease than untreated pig skin samples. The reason is as discussed for rat skin.

#### Macroscale friction and effect of velocity, normal load effect and number of cycles

**Figure 14:**
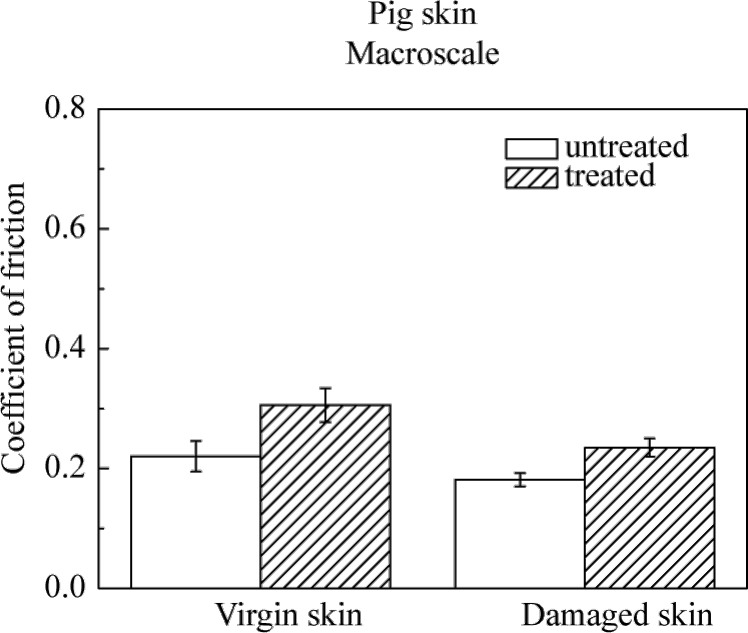
Coefficient of friction on macroscale for virgin pig skin, damaged skin, treated virgin skin and treated damaged skin.

**Figure 15:**
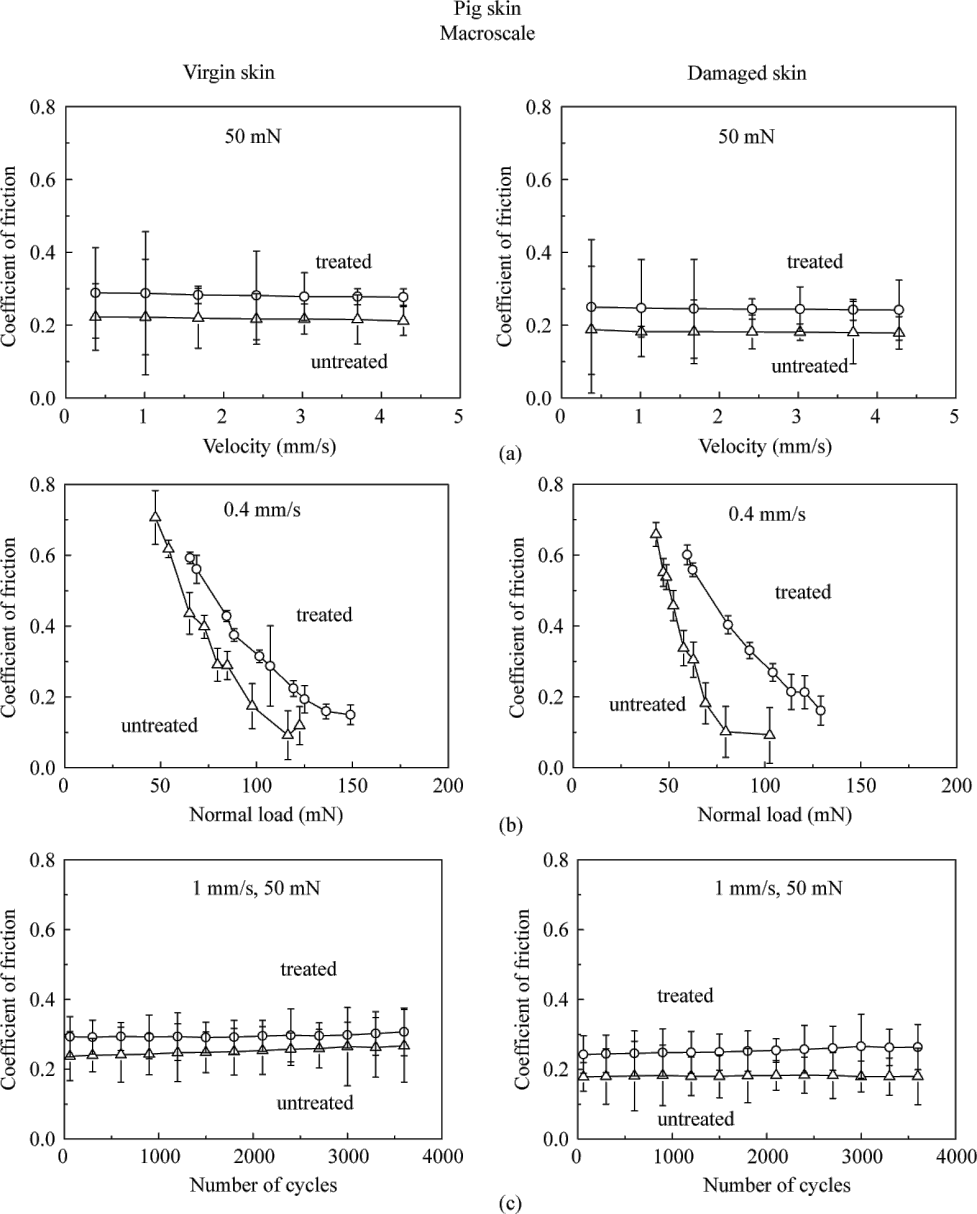
Effect of (a) velocity, (b) normal load and (c) number of cycles on the coefficient of friction on the macroscale for virgin pig skin, damaged skin, treated virgin skin and treated damaged skin.

Figure 14 shows the coefficient of friction of the four pig-skin samples on the macroscale. Trends and the values of the coefficient of friction are similar to those of the rat skin. Figure 15 shows the effect of velocity, normal load, and number of cycles on the macroscale. The coefficient of friction does not show a significance change with the increasing velocity. The coefficient of friction decreases as the normal load increases. The coefficient of friction remains constant with an increase in the number of cycles. Again trends are similar to those for the rat skin.

## Conclusion

In this study, rat skin and pig skin were used as experimental samples to study their friction properties and durability. The effect of velocity, normal load, and relative humidity on the coefficient of friction for virgin skin, cream-treated virgin skin, damaged skin and cream-treated damaged skin were studied on the nano- and macroscale. The durability of skin samples was also studied by repeated cycling tests.

The virgin rat skin has lower nanohardness and elastic modulus than that of virgin pig skin, and that of the damaged skin is higher than that of the virgin skin for both rat and pig skin. For rat skin, damaged skin has a larger roughness than virgin skin. After treatment with skin cream, the roughness decreases. The contact angle value of virgin skin is lower than damaged skin. The contact angle decreases after treatment due to an increasing hydrophilicity. Skin cream increases the hydrophilic properties of the skin. For pig skin, the roughness shows a similar trend to that of rat skin, but the contact angle of damaged skin shows a significant increase as compared to virgin skin because of the reduction in lipids present on the surface.

On the nanoscale, for both rat and pig skin samples, the coefficient of friction of damaged skin is larger than that of virgin skin. After treatment, the coefficient of friction increases because of meniscus formation. The effect of velocity on the nanoscale coefficient of friction of rat and pig skin samples shows the same trend. When the velocity increases, the coefficient of friction decreases. For untreated skin it is because the meniscus force cannot be fully reformed during sliding, but for treated skin it is because the viscosity decreases with the increasing shear rate. At the beginning as the normal load increases, the coefficient of friction decreases for rat and pig skin on the nanoscale, because the tip does not slide in a steady manner on the surface at low normal load, leading to a high deflection of the tip. After a certain value of the normal load, when the normal load further increases, the coefficient of friction of untreated skin samples remains constant, while that of treated skin samples shows a slightly increase due to an increasing meniscus force. As the relative humidity increases, the coefficient of friction of untreated rat and pig skin on the nanoscale does not increase much, because the water hardly absorbs or penetrates into skin surface because of a thin hydrophobic lipid layer. For the treated skin samples, the humectants help the skin surface to attract water molecules, which increase the adhesive force and coefficient of friction.

The coefficient of friction does not show a significant change in the durability tests. The coefficient of friction on the macroscale is larger than on the nanoscale. On macroscale, the coefficient of friction of damaged skin is comparable to that of virgin skin. After treatment, the coefficient of friction increases compared with untreated skin samples because of the formation of meniscus bridges. On macroscale, the velocity and number of cycles do not have an obvious effect on the coefficient of friction. When the normal load increases, the coefficient of friction decreases due to an increased surface roughening and a large quantity of wear debris. The coefficient of friction of pig skin is larger than that of rat skin on the nanoscale. The effect of velocity, normal load, and relative humidity on pig skin has the same trend as that for rat skin both on the nano- and macroscale, as does the durability. The differences of friction properties between the four skin samples on pig skin are more distinct than those of the rat skin samples.

This research demonstrates that skin cream can smooth the skin surface and increase the hydrophilic properties of skin. The damaged skin surface condition can be improved by skin cream. The coefficient of friction of skin depends on the velocity, normal load, relative humidity and number of cycles.
